# The Time-varying Impact of Pancreas Graft Failure on Mortality and Kidney Graft Outcomes in Simultaneous Pancreas and Kidney Transplantation

**DOI:** 10.1097/TXD.0000000000001942

**Published:** 2026-06-02

**Authors:** Christie Rampersad, Jeffrey Schiff, Chaya Shwaartz, S. Joseph Kim

**Affiliations:** 1 Division of Nephrology and the Ajmera Transplant Centre, University Health Network, Univesity of Toronto, Toronto, ON, Canada.; 2 Institute of Health Policy, Management and Evaluation, Dalla Lana School of Public Health, University of Toronto, Toronto, ON, Canada.; 3 Department of Surgery, University Health Network, University of Toronto, Toronto, ON, Canada.

## Abstract

**Background.:**

Simultaneous pancreas-kidney transplantation improves survival and glycemic control in insulin-dependent diabetes, but pancreas graft failure remains common, and its evolving impact on patient survival and downstream kidney outcomes is inadequately defined.

**Methods.:**

Using 2013–2022 US Scientific Registry of Transplant Recipients data, we treated pancreas death-censored graft failure (DCGF) as a time-dependent exposure in Cox models for mortality, and kidney all-cause graft failure and DCGF. Early (≤1 y) and landmark (>1 y) analyses addressed nonproportional hazards. Sensitivity analyses conducted on post-March 2018 standardized failure definitions and technical failures. Subgroup analyses analyzed age, sex, and diabetes type. In a 1-y landmark subcohort with intact grafts, we analyzed associations between subsequent kidney DCGF and 5-y mortality.

**Results.:**

Pancreas DCGF occurred in 1013 recipients (5-y incidence 13.7%), predominantly within 1 y (33% technical), conferring increased mortality early (hazard ratio [HR] 2.97; 95% confidence interval [CI], 2.04-4.34) and sustained risk beyond 1 y (HR 2.47; 95% CI, 1.87-3.25), with similar findings post-2018. Technical DCGF carried modest risk (HR 1.56; 95% CI, 1.16-2.09). Pancreas DCGF was also associated with kidney all-cause graft failure (HR 2.33; 95% CI 2.01, 2.69) and DCGF (HR 2.70; 95% CI, 2.25-3.25). In those with intact grafts at 1 y, kidney DCGF conferred markedly higher 5-y mortality (HR 9.37; 95% CI, 7.04-12.48). Younger recipients exhibited slightly greater relative mortality risk after pancreas DCGF (ratio of HR 1.10).

**Conclusions.:**

Pancreas DCGF is common and independently heralds substantially increased risks of death and kidney graft loss, whereas kidney failure further amplifies mortality, underscoring the need for enhanced risk stratification, prevention, and post–failure management to optimize long-term simultaneous pancreas-kidney outcomes. Younger recipients also faced a higher relative mortality risk after pancreas failure, highlighting a vulnerable subgroup requiring further study.

## INTRODUCTION

Simultaneous pancreas-kidney (SPK) transplantation is the preferred treatment for select patients with insulin-dependent diabetes and kidney failure, offering improved glycemic control, metabolic function, and quality of life.^1,2^ Compared with kidney transplant alone, SPK is associated with superior long-term survival, with 5-y patient survival rates ~90%.^3^ Despite these benefits, pancreas graft failure remains a persistent challenge, affecting >25% of recipients within 5 y.^2,3^ Early failures are primarily technical, most commonly due to vascular thrombosis or duodenal leak, whereas late failures are largely due to chronic rejection or death with graft function.^2-5^ The consequences of pancreas graft failure extend beyond the loss of endocrine function, as evidence suggests it may accelerate kidney graft failure and increase mortality risk.^6,7^ However, a more nuanced examination of adverse outcomes after pancreas graft failure is warranted to understand the distinct failure mechanisms associated with specific risk profiles, the interrelationship between organ failures, and the time-varying risk of mortality.^5^

Previous studies on the impact of pancreas graft failure on subsequent kidney graft failure have yielded mixed results, reflecting differences in study design and analytic approach. Two US registry studies found higher kidney failure risk after pancreas loss—one (1997–2005) reported a 64% increased risk but excluded early kidney failures, whereas another (2000–2016) in 641 recipients with type 2 diabetes estimated a 74% increased risk in a smaller selected cohort. Although these studies used landmark approaches to partially mitigate immortal time bias, classification within the first year posttransplant nonetheless required survival to the landmark and was therefore less sensitive to immediate adverse effects of graft failure, may have preferentially excluded early occurring technical pancreas failures, and may have incompletely captured later-occurring failures, with potential attenuation of risk estimates.^6,7^ Although improved kidney graft survival after pancreas retransplantation has been observed, this indirect evidence is potentially limited by strong confounding and selection biases affecting which patients were retransplanted.^8^ The extent to which pancreas graft failure contributes to kidney failure remains uncertain, necessitating further investigation.

The impact of graft survival on mortality is a key concern for patients, providers, and healthcare systems. In kidney transplant recipients, graft failure confers a substantial mortality risk, particularly in those with diabetes and in the early post–failure period.^9-12^ Although similar patterns have been suggested in SPK recipients, prior studies vary in design and analytic approach. Four registry studies (Scientific Registry of Transplant Recipients [SRTR], International Pancreas Transplant Registry, 1988–2007) reported hazard ratios (HRs) for mortality of 10.38 to 14.99 after kidney failure and 2.6 to 5.64 after pancreas failure, with one estimating 85% and 15% reduction in expected posttransplant survival, respectively. However, these older cohorts spanned multiple transplantation eras, had minimal confounder adjustment, inconsistently reported effect estimates, and were prone to immortal time bias, whereas 2 studies used a 1-y landmark.^6,13-15^ A more recent SRTR analysis (2000–2016) reported a 3-fold higher mortality risk in a highly selected cohort (n = 40) with pancreas failure at 3 mo posttransplant.^7^ To mitigate immortal time bias present in prior studies, Gruessner et al (SRTR 2001–2016) used time-dependent modeling and estimated 10- and 2.6-fold higher mortality after kidney and pancreas failures, respectively. However, requiring 6 mo of follow-up excluded early posttransplant deaths, again introducing potential selection bias.^5^ Additionally, all prior studies predate the standardized Organ Procurement and Transplantation Network (OPTN) pancreas graft failure definition implemented in 2018, further complicating interpretation in a contemporary context.^5-7,13-15^

To address these gaps, we conducted a contemporary study of SPK recipients in a large US registry cohort to evaluate the time-varying risk of mortality and kidney graft failure after pancreas graft failure.

## MATERIALS AND METHODS

### Study Design and Population

This cohort study consisted of all consecutive deceased donor SPK transplants performed in the United States from January 1, 2013, to December 31, 2022. Patients were eligible for inclusion if they had not received a prior solid organ transplant. This study used data from the SRTR. The SRTR data system includes data on all donor, waitlisted candidates, and transplant recipients in the United States, submitted by the members of the OPTN. The Health Resources and Services Administration, US Department of Health and Human Services, provides oversight to the activities of the OPTN and SRTR contractors. Data are mandatorily reported by transplant centers at the time of donation and transplantation, and with posttransplant follow-up records at 6 mo, 1 y, and then annually, until the recipient is retransplanted, dies, or is lost to follow-up. The study was approved by the University Health Network Research Ethics Board, and the need for written informed consent was waived.

### Outcomes

The primary outcome of interest was all-cause mortality, with recipients censored at last follow-up. Secondary outcomes were kidney death-censored kidney graft failure (DCGF) and all-cause graft failure (ACGF), as time-to-event outcomes. We also reported a contemporary epidemiologic description of pancreas graft failure, including cumulative incidence, posttransplant timing, sequence of graft failure, and cause.

### Graft Failure Exposures

The primary exposure was pancreas DCGF as a time-dependent predictor of mortality and kidney graft failure. Pancreas graft failure was captured in the SRTR registry based on reporting by transplant centers, with uniform definitions proposed by the OPTN Pancreas Transplantation Committee implemented in February 2018: Pancreas graft failure was defined by any of (1) the transplanted pancreas is removed; (2) a recipient re-registers for a pancreas transplant; (3) a recipient registers for an islet transplant after undergoing pancreas transplant; (4) a recipients dies; or (5) a recipient has total insulin use of ≥0.5 units/kg/d for 90 consecutive days.^16^

A secondary analysis also considered a landmarked cohort with functioning kidney and pancreas allografts at 1 y posttransplant and analyzed subsequent kidney DCGF as a time-dependent predictor of mortality, irrespective of pancreas graft status.

In time-dependent analyses, recipients initially contributed follow-up time for functioning grafts. If pancreas or kidney graft failure occurred, subsequent follow-up time was reclassified to reflect the post–failure period. Outcomes were attributed to the graft status present at the time the event occurred.

### Statistical Analysis

Analyses were conducted using R Studio (version 2023). Descriptive statistics were performed with categorical variables presented as frequencies or percentages and compared with the chi-square test. Continuous variables were presented as the mean with SD and compared using the Student’s *t* test.

### Epidemiologic Description of Pancreas DCGF

The cumulative incidence of pancreas DCGF was visualized overall and reported at 5 y posttransplant. To assess the potential impact of changing pancreas graft failure definitions on reporting practices, the cumulative incidence of pancreas DCGF was also plotted for recipients; transplanted pancreas DCGF events were reported by year posttransplant. Recipients were also characterized by whether they experienced pancreas DCGF or kidney DCGF; among those who experienced both organ failures, the sequence of failure events was characterized as simultaneous (if the grafts failed within 1 y of each other), or whichever organ failed first. Causes of pancreas graft failure were also reported and categorized as technical (surgical complications such as bleeding, thrombosis, anastomotic leak), nontechnical (rejection, infection), or unknown (nonspecific pancreatitis, or unspecified).

### Survival Analyses

Multivariable Cox proportional hazards models were used to assess the association between time-dependent pancreas DCGF and mortality while adjusting for potential confounders. Pancreas DCGF was modeled as a time-dependent exposure to ensure that recipients contributed person-time to the unexposed group until graft failure occurred, thereby minimizing immortal time bias that can arise with standard Cox or landmark-based approaches in observational time-to-event analyses.^17^ Covariables were selected a priori, and causal relationships were visualized in a directed acyclic graph (**Figure S1, SDC,** https://links.lww.com/TXD/A869). Potential covariables included characteristics of the donors (age, biologic sex, race, body mass index, stroke as cause of death, donation after circulatory death status, terminal serum creatinine), recipients (age at transplant, biologic sex, race, diabetes type, weight, height, dialysis status, wait time on dialysis, history of malignancy), donor-recipient pairing (number of whole antigen HLA matches, presence of cytomegalovirus serologic mismatch with donor seropositive-recipient seronegative), and transplants (delayed graft function, and induction and maintenance immunosuppression). All variables had <10% missingness; the mechanism was assumed to be random and simple imputation was used. The latter involved median substitution for continuous variables and a random number generated on the basis of the distribution of cumulative percentages for categorical variables.

The proportional hazards assumption was assessed using the Grambsch-Therneau test and visually inspected with Schoenfeld residual plots over time. HRs were presented with their 95% confidence intervals (CIs). Because the proportionality assumption was violated in the model for pancreas DCGF-related mortality, the analysis was done in 2 parts: (1) the “early cohort” analyzed outcomes up to 1 y posttransplant, and otherwise censored at 1 y, followed by (2) analysis of a “landmarked cohort” consisting of recipients with functioning pancreas allografts landmarked at 1 y posttransplant. Among recipients with pancreas DCGF, a Kaplan-Meier curve was generated from the time of graft failure to assess variation in post–failure mortality risk.

Multivariable Cox proportional hazards models were similarly used to evaluate associations between time-dependent pancreas DCGF and kidney outcomes of ACGF and DCGF and between time-dependent kidney DCGF and mortality.

As an exploratory analysis, causes of death were compared between recipients with and without pancreas graft failure using SRTR-reported cause-of-death categories. Distributions were compared using the chi-square test, with causes grouped into cardiovascular, infection, malignancy, graft failure, hemorrhage, external/behavioral, other medical, and unknown/other.

### Sensitivity Analysis

To account for potential measurement error introduced by inconsistent pancreas failure definitions, the survival analysis of pancreas DCGF as a time-dependent predictor of mortality was repeated in a subcohort of recipients transplanted after March 1, 2018, following the introduction of a standardized pancreas graft failure definition by the OPTN.

We conducted an additional analysis of the time-dependent mortality risk from technical causes of pancreas DCGF. In this analysis, patients were censored at the time of nontechnical pancreas DCGF or last follow-up. The analysis of kidney DCGF-related mortality was similarly assessed in a landmarked cohort with functioning kidney allografts at 1 y, and outcomes were assessed up to 5 y posttransplant.

### Subgroup Analysis

Two-way interactions were tested between pancreas DCGF and each of recipient age, biologic sex, and diabetes type (type 1 versus 2).

As a data-driven exploratory analysis of effect measure modification by recipient age, the HR for pancreas DCGF-associated mortality was plotted across age ranges and K-means clustering with the elbow method was used to identify the optimal number of age-group clusters. Univariable Cox proportional hazards models for mortality were conducted in dichotomized subgroups of recipients based on age thresholds. When the HRs for subgroups were significant, the ratio of HRs was reported.

A 2-sided *P* value of <0.05 was considered statistically significant.

## RESULTS

### Study Population

The analytic cohort consisted of 7677 SPK transplant recipients with 40 542 person-years of follow-up. The mean recipient age was 42.2 ± 9.2 y and 62% were male. Additional baseline characteristics of the analytic cohort are shown in Table 1. Missingness across covariates was minimal, with most variables <1% and all below a prespecified 10% threshold.

**TABLE 1.** Baseline demographics of SPK recipients with and without death-censored pancreas graft failure, with standardized mean differences

Characteristics	Distribution
Donor characteristics	
Age, y	24.4 (8.1)
Male sex, n (%)	5368 (69.9%)
White race, n (%)	5809 (75.7%)
Black race, n (%)	1594 (20.8%)
Weight, kg	71.2 (15.0)
Height, cm	172.2 (11.8)
Body mass index, kg/m^2^	23.9 (3.9)
Peridonation characteristics	
Stroke as cause of death, n (%)	746 (9.7%)
Donation after circulatory death, n (%)	227 (3.0%)
Cold ischemic time, h	10.4 (4.6)
Terminal serum creatinine, mg/dL	0.95 (0.57)
Terminal serum creatinine >1.5 mg/dL, n (%)	597 (7.8%)
Recipient characteristics	
Age at transplant, y	42.2 (9.2)
Male sex, n (%)	4733 (61.7%)
ABO, n (%)	
A	2675 (34.8%)
B	953 (12.4%)
AB	293 (3.8%)
O	3756 (48.9%)
White race, n (%)	5129 (66.8%)
Black race, n (%)	2148 (28.0%)
Weight, kg	75.1 (14.9)
Height, cm	170.6 (10.8)
Type 1 diabetes, n (%)	6187 (80.6%)
Wait time on dialysis, d	328.1 (412.9)
CMV seropositive, n (%)	4183 (54.9%)
EBV seropositive, n (%)	6614 (86.2%)
Donor-recipient characteristics	
No. of HLA whole antigen mismatches	4.63 (1.1)
CMV mismatch (%)	1650 (21.5%)
Transplant center characteristics	
SPK transplant volume quartile, n (%)	
Q1 (lowest volume)	315 (4.1%)
Q2	988 (12.9%)
Q3	1950 (25.4%)
Q4	4424 (57.6%)
Peritransplant complications	
Delayed graft function, n (%)	673 (8.8%)

CMV, cytomegalovirus; EBV, Epstein-Barr virus; SPK, simultaneous pancreas and kidney transplant.

**FIGURE 1.**
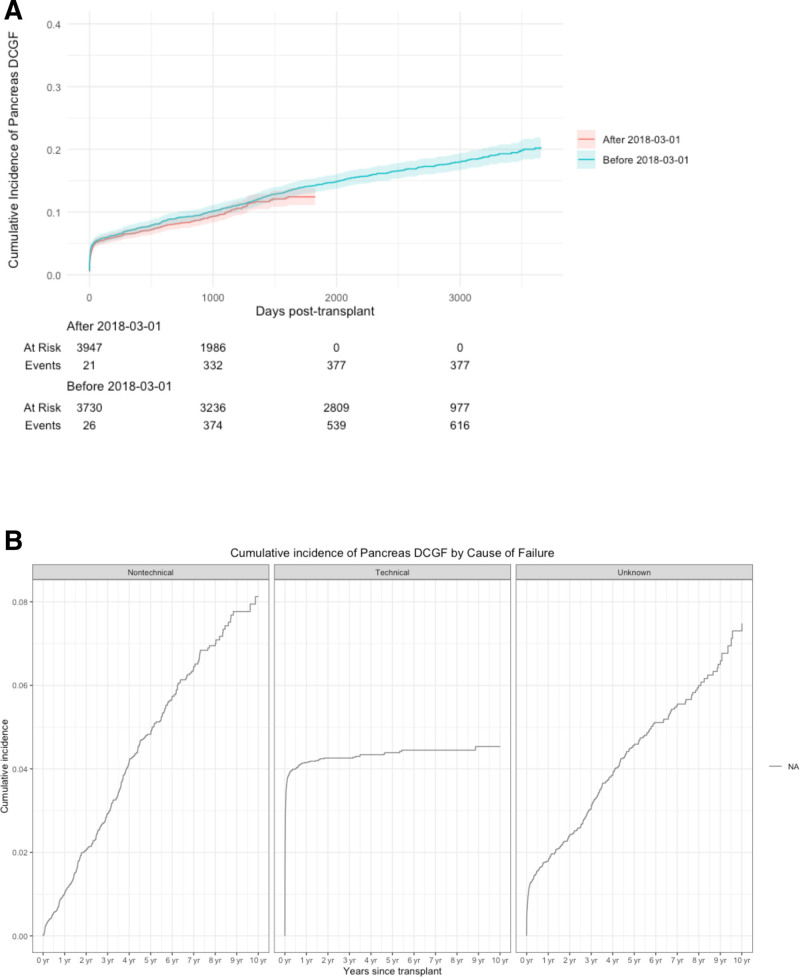
Cumulative incidence of pancreas death-censored graft failure. A, Cumulative incidence of pancreas DCGF for recipients transplanted before and after March 1, 2018 (*P* = 0.1). B, Cumulative incidence of pancreas DCGF by technical vs nontechnical vs unknown causes. DCGF, death-censored graft failure.

Pancreas DCGF occurred in 1013 recipients, with a 5-y cumulative incidence of 13.7%. The incidence was unchanged by the introduction of a standardized failure definition (Figure 1A; **Figure S2, SDC,** https://links.lww.com/TXD/A869). The majority of pancreas failures occurred in the first year, and predominantly in the first month posttransplant (**Figure S3, SDC,** https://links.lww.com/TXD/A869). Early failures were dominated by technical causes (n = 334) including graft/vascular thromboses (n = 282), whereas nontechnical failures (n = 352) from rejection or infection tended to occur later, and the cause was unspecified in 327 cases (**Table S1, SDC,** https://links.lww.com/TXD/A870; Figure 1B).

Although most recipients experienced no graft loss (6384/7677; 83%), pancreas failure alone occurred in 681 (9%), kidney failure alone occurred in 280 (4%), and both grafts failed in 332 recipients (4%). Among cases where both grafts failed, the majority of these occurred within 1 y of each other (n = 210; **Figure S4, SDC,** https://links.lww.com/TXD/A869).

### Pancreas DCGF-associated Mortality

In the early cohort, there were 528 pancreas DCGF and 205 deaths, whereas the landmarked cohort had 485 pancreas DCGF and 515 deaths. The development of pancreas DCGF was associated with an increased relative hazard for mortality both at 1 y (HR 2.97 [95% CI, 2.04-4.34]) and beyond 1 y (HR 2.47 [95% CI, 1.87-3.25]) posttransplant. Similar relative hazards for mortality were seen in the 2018 subcohort (Table 2). Kaplan-Meier analysis showed a steady decline in survival after pancreas DCGF, without a sharp inflection over time (**Figure S5, SDC,** https://links.lww.com/TXD/A869).

**TABLE 2.** Multivariable Cox proportional hazards models for mortality and kidney failure outcomes in SPK transplant recipients, where pancreas DCGF events were considered as a time-dependent predictor (N = 7677 recipients)

Outcome	Model	No. of patients	No. of events	HR (95% CI)	*P*
Mortality	Full follow-up^*a*^	8690	769	1.98 (1.65-2.39)	**<0.001**
	1-y survival	8205	205	2.98 (2.04-4.36)	**<0.001**
	>1-y landmark	7412	515	2.43 (1.85-3.20)	**<0.001**
	Technical pancreas DCGF	8011	672	1.57 (1.17-2.10)	**0.003**
	2018 cohort	4324	247	2.76 (1.97-3.86)	**<0.001**
All-cause kidney graft failure	Full follow-up	8498	1319	2.33 (2.01-2.69)	**<0.001**
Kidney DCGF	Full follow-up	8498	729	2.70 (2.25-3.25)	**<0.001**

Note that there was a total of 7677 recipients in the cohort. In this time-dependent analysis by pancreas DCGF status, patients were therefore represented more than once in the cohort based on before and after development of pancreas DCGF. Pancreas DCGF events in the early cohort <1 y posttransplant: 528. Pancreas DCGF events in the landmarked cohort >1 y posttransplant: 485.

Bold values denote statistical significance.

The models were adjusted for donor characteristics (age, sex, race, body mass index, stroke as cause of death, donation after circulatory death status, terminal serum creatinine), recipient characteristics (age at transplant, sex, race, diabetes type, wait time on dialysis, weight, height, history of malignancy), donor-recipient characteristics (number of whole antigen HLA mismatches, CMV mismatch status), peritransplant characteristics (cold ischemic time, induction immunosuppression, maintenance immunosuppression (steroid, calcineurin inhibitor, mycophenolic acid), and kidney delayed graft function status (defined by dialysis within 1 wk posttransplant)).

^*a*^Cox proportional hazard assumption violated.

CI, confidence interval; CMV, cytomegalovirus; DCGF, death-censored graft failure; HR, hazard ratio; SLK, simultaneous liver-kidney transplantation.

**FIGURE 2.**
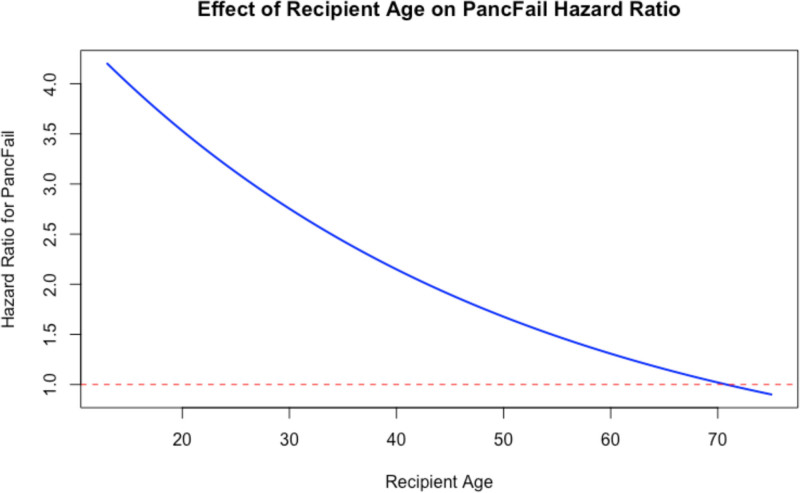
Plot of hazard ratio for pancreas DCGF-related mortality across range of recipient ages. DCGF, death-censored graft failure.

The prespecified subgroup analyses revealed a significant interaction between pancreas DCGF and recipient age at the time of transplant, with the HR for pancreas DCGF nonlinearly decreasing with increasing age. When age was instead summarized into broad, data-driven clusters, the average subgroup-specific HRs were similar, reflecting aggregation across wide age ranges rather than the absence of age-related heterogeneity (Figure 2; **Figure S6, SDC,** https://links.lww.com/TXD/A869). We also observed increased mortality risk when pancreas DCGF occurred beyond 1 y for recipients with type 1 (HR 2.74 [95% CI, 2.05-3.66]; n = 6020 recipients, 417 deaths) but not type 2 diabetes (HR 1.04 [95% CI, 0.40-2.68]; n = 1392 recipients, 98 deaths; ratio of HR 2.63). There was no interaction between pancreas DCGF and biologic sex.

When pancreas DCGF was restricted to only technical causes (n = 334), its development was associated with a more modest increase in the relative hazard for mortality (HR 1.56 [95% CI, 1.16-2.09]; Table 2). Among patients with technical pancreas DCGF, the majority (85%) were alive at the end of the study period, and 85% of the deaths in this group occurred >1 mo after graft failure.

The distribution of reported causes of death did not differ significantly between recipients with and without pancreas graft failure (*P* = 0.21), with a substantial proportion of deaths classified as unknown or other in both groups (~43%–44%; **Table S2, SDC,** https://links.lww.com/TXD/A870).

### Pancreas DCGF-associated Kidney Graft Failure

There were 1319 kidney ACGF and 729 kidney DCGF events during the follow-up period. Pancreas DCGF was associated with subsequently higher risks of both kidney ACGF (HR 2.33 [95% CI, 2.01-2.69]) and DCGF (HR 2.70 [95% CI, 2.25-3.25]; Table 2).

### Kidney DCGF-associated Mortality

Among 6856 recipients with functioning kidney and pancreas allografts at 1 y, subsequent kidney DCGF (n = 544 events) was associated with marked 5-y mortality (HR 9.37 [95% CI, 7.04-12.48]).

## DISCUSSION

In this contemporary registry cohort of 7677 SPK transplant recipients with >40 000 person‐years of follow-up, we observed significant and dynamic risks of adverse outcomes after pancreas graft failure. Pancreas graft failure was associated with nearly 3-fold higher mortality risk within the first year posttransplant and a sustained elevated risk beyond 1 y, with effects more pronounced in younger recipients. Moreover, technical failures resulted in a more modest yet significant mortality risk. Pancreas graft failure was also linked to >2-fold increased risk for both all-cause and DCGF, and kidney failure conferred almost a 9-fold higher 5-y mortality risk. These findings underscore the complex, time-varying relationships between pancreas and kidney graft outcomes in SPK recipients. Although SPK transplantation is inherently a dual-organ procedure, we centered this analysis on pancreas graft failure to clarify its independent prognostic significance within contemporary SPK recipients. By using time-dependent modeling in a national cohort spanning 2013 to 2022, we were able to capture both early and late risks of pancreas failure without immortal-time bias, producing risk estimates that reflect contemporary practice and the standardized OPTN definitions adopted in 2018.

Our findings highlight the association between pancreas graft failure and mortality risk in SPK transplant recipients, with affected patients facing a 2–3 times higher mortality compared with those with functioning grafts. Prior studies reported similar effect estimates but faced potential limitations of immortal time bias or shortcomings of landmark analysis.^6,7,13-15^ To address these issues, we treated pancreas graft failure as a time-dependent exposure, classifying patients as having graft failure only after its occurrence. We then divided our cohort into an early cohort and a 1-y landmarked cohort to account for nonproportional hazards. Technical and nontechnical failure causes exhibited distinct cumulative incidence profiles; thus, the immediate effects of technical failures were expected to be captured in the early cohort, whereas the long-term effects of nontechnical failures were represented in the landmark cohort. Importantly, even technical failures, which might be assumed to carry more immediate perioperative risks, were associated with a modest but sustained increase in mortality. Most of these deaths occurred >1 mo after graft loss, suggesting that patients were not dying directly from the surgical complication itself, but rather from longer-term downstream effects. This distinction underscores the need for structured post–failure management and long-term follow-up. That effect estimates were similar to prior studies likely reflects the early timing of many pancreas graft failures, particularly technical failures, which conferred modest mortality risks, but the use of time-dependent modeling ensures appropriate attribution of risk across both early and late failure phenotypes without reliance on arbitrary landmarks.

To date, only the 2018 study by Gruessner et al^5^ used time-dependent modeling, examining the effects of nontechnical failures. Our study supports their findings with comparable effect estimates while extending them to a more contemporary cohort reflective of current clinical practice and incorporating standardized definitions of pancreas graft failure. Moreover, by not requiring a minimum follow-up period, we were able to assess the early impact of technical failures—typically occurring within the first 1 to 3 mo posttransplant—on mortality, a novel finding. We also observed that younger recipients experienced a greater relative hazard of mortality than older recipients after pancreas graft failure. Although younger patients might be expected to tolerate graft loss better, this finding suggests a paradoxical vulnerability that merits further exploration. Possible contributors include heightened immune reactivity, nonadherence, or cardiovascular complications in long-standing type 1 diabetes. Importantly, whether pancreas retransplantation can mitigate this excess risk remains uncertain and represents an important area for future investigation.^18^ Increased mortality risk after pancreas failure among recipients with type 1 diabetes may reflect their younger age, longer disease duration, and greater vulnerability to cardiovascular complications.^19,20^ The absence of a similar association in recipients with type 2 diabetes may reflect either limited power in this smaller subgroup or a true difference in how graft failure influences mortality risk, requiring further studies.

Our analysis further indicates that pancreas graft failure has significant downstream consequences on kidney graft outcomes. Our study, using a contemporary registry cohort of 7677 SPK transplant recipients and time-dependent modeling, demonstrates that patients experiencing pancreas graft failure face over a 2-fold increased hazard for subsequent kidney graft failure. To provide clinical context for interpreting the mortality risk observed after pancreas graft failure, we also analyzed mortality after kidney graft failure, a well-recognized high-risk event in SPK recipients. Importantly, for kidney failure-associated mortality, we reaffirm the findings of the Gruessner study, with kidney graft failure itself conferring nearly a 9-fold higher 5-y mortality risk.^5^ This risk is relatively higher than the mortality risk seen after graft failure in kidney transplant alone.^11^ Considered together, these findings help situate the magnitude and clinical significance of pancreas DCGF-associated mortality. These observed associations also suggest an indirect pathway in which pancreas graft failure may contribute to mortality through its impact on kidney function. Although the precise causal mechanism remains uncertain, potential contributors include shared alloimmune injury, impaired glycemic control precipitating diabetic kidney injury, and alterations in immunosuppressive management during the transitional period. Additional factors, such as complications arising from surgical management of the failed pancreas graft, or the sequelae of immunosuppression (including infections, malignancy, and cardiovascular events), may further compound the risk. Although we did not formally implement a multistate model, the kidney DCGF-mortality analysis supports a plausible mechanistic link in the interdependent pancreas-kidney outcomes observed in SPK transplantation. Future studies using multistate modeling and incorporating granular clinical data are warranted to definitively delineate these interrelated pathways and clarify whether interventions, including pancreas retransplantation, can modify this heightened mortality risk.

Although identifying these risks is valuable for patient counseling and care planning, our findings underscore the need for enhanced upstream risk stratification and standardized clinical guidelines. Currently, available prediction tools for pancreas graft failure remain limited.^21,22^ The pancreas donor risk index is the only externally validated risk index for solid pancreas transplantation; however, it incorporates only donor factors, is associated solely with short-term (1 y) graft survival, exhibits modest discrimination (c-statistic 0.67) with no calibration reported, and has shown poor generalizability in many jurisdictions.^21,23-29^ Given the various phenotypes of pancreatic failure, a prediction model for technical failure derived from a single-center cohort spanning 1998 to 2011 demonstrated poor predictive performance (c-statistic 0.52–0.60) and has not been externally validated.^30^ Enhanced risk stratification tools integrating both donor and recipient factors could more accurately identify patients at the highest risk, thereby enabling targeted interventions to prevent pancreas graft failure and mitigate its downstream effects on kidney graft function and overall mortality.

Once a pancreas graft has failed, there is a pressing need for clear post–failure management protocols as this group faces markedly increased risks of adverse outcomes.^31^ Although the subject of reviews, there is a need for consensus guideline recommendations addressing outcomes and management after pancreas graft failure to provide standardized approaches for immunosuppression weaning, surgical management, and decisions regarding pancreas retransplantation.^32-35^ This gap is especially critical for lower-volume centers or when patients transition care after graft failure. These issues highlight the imperative for comprehensive, evidence-based approaches to both risk prediction and post–failure management, which are essential for optimizing long-term outcomes in SPK recipients.

Our study benefited from a large, contemporary national registry cohort of 7677 SPK recipients spanning 2013 to 2022, with >40 000 person-years of follow-up. The use of time-dependent modeling to treat pancreas graft failure as an exposure, coupled with consideration of the effects of different failure phenotypes, enabled a comprehensive and dynamic assessment of the impact on both mortality and kidney graft outcomes while mitigating immortal time bias.^17^ In addition, the availability of detailed donor, recipient, and peritransplant variables allowed for robust adjustment of potential confounders and facilitated subgroup analyses, such as those evaluating the modifying effects of recipient age and diabetes type. Our results were robust to sensitivity analyses accounting for potential measurement error and benefited from the implementation of standardized definitions of pancreas graft failure during the study period.

Despite these strengths, our findings are subject to residual unmeasured confounding and potential misclassification biases, given the observational, registry-based nature of the study cohort. Second, pancreas graft failure dates are reported by centers and may be incomplete or variably ascertained; missing dates were handled as censoring at last follow-up, but residual misclassification remains possible. The quality and granularity of the registry data are inherently limited; for example, detailed clinical, histologic, or immunologic information regarding causes of graft failure was not available.^36,37^ Moreover, although our cohort reflects current US clinical practice, the results may not be fully generalizable to other regions or transplant populations with differing practices and patient profiles. Future studies integrating more granular clinical data and using multistate modeling approaches are needed to refine risk stratification and elucidate the causal pathways underlying observed associations. Future studies should also analyze how outcomes after pancreas graft failure compare with those of kidney-alone transplant recipients. Finally, further studies should assess patient-centered outcomes such as quality of life, which is known to be impaired after kidney graft failure but has not yet been explored in pancreas transplantation.^38^

In conclusion, these findings identify pancreas graft failure in SPK recipients as a pivotal event, linked to markedly worse outcomes. Pancreas graft failure was common and associated with 2- to 3-fold higher risks of both mortality and kidney graft failure. Notably, kidney failure was further correlated with nearly a 9-fold increase in 5-y mortality. Younger recipients were particularly vulnerable. These results underscore the interdependent relationship between pancreas and kidney outcomes and highlight the urgent need for improved risk stratification and standardized postfailure management.

## ACKNOWLEDGMENTS

The data reported here have been supplied by the Hennepin Healthcare Research Institute as the contractor for the SRTR. The interpretation and reporting of these data are the responsibility of the author(s) and in no way should be seen as an official policy of or interpretation by the SRTR or the US Government.

## Supplementary Material


