# Supplementary material for: The Time-varying Impact of Pancreas Graft Failure on Mortality and Kidney Graft Outcomes in Simultaneous Pancreas and Kidney Transplantation
Source: Transplant Direct. 2026 Jun 2;12(7):e1942. doi: 10.1097/TXD.0000000000001942 (PMC13232909; doi:10.1097/TXD.0000000000001942)
Supplement: Supplementary file 1 [file txd-12-e1942-s001.pdf]

Figure S1. Directed acyclic graph

Directed acyclic graph representing assumed causal relationships among donor, recipient, perioperative factors, graft failure, and mortality

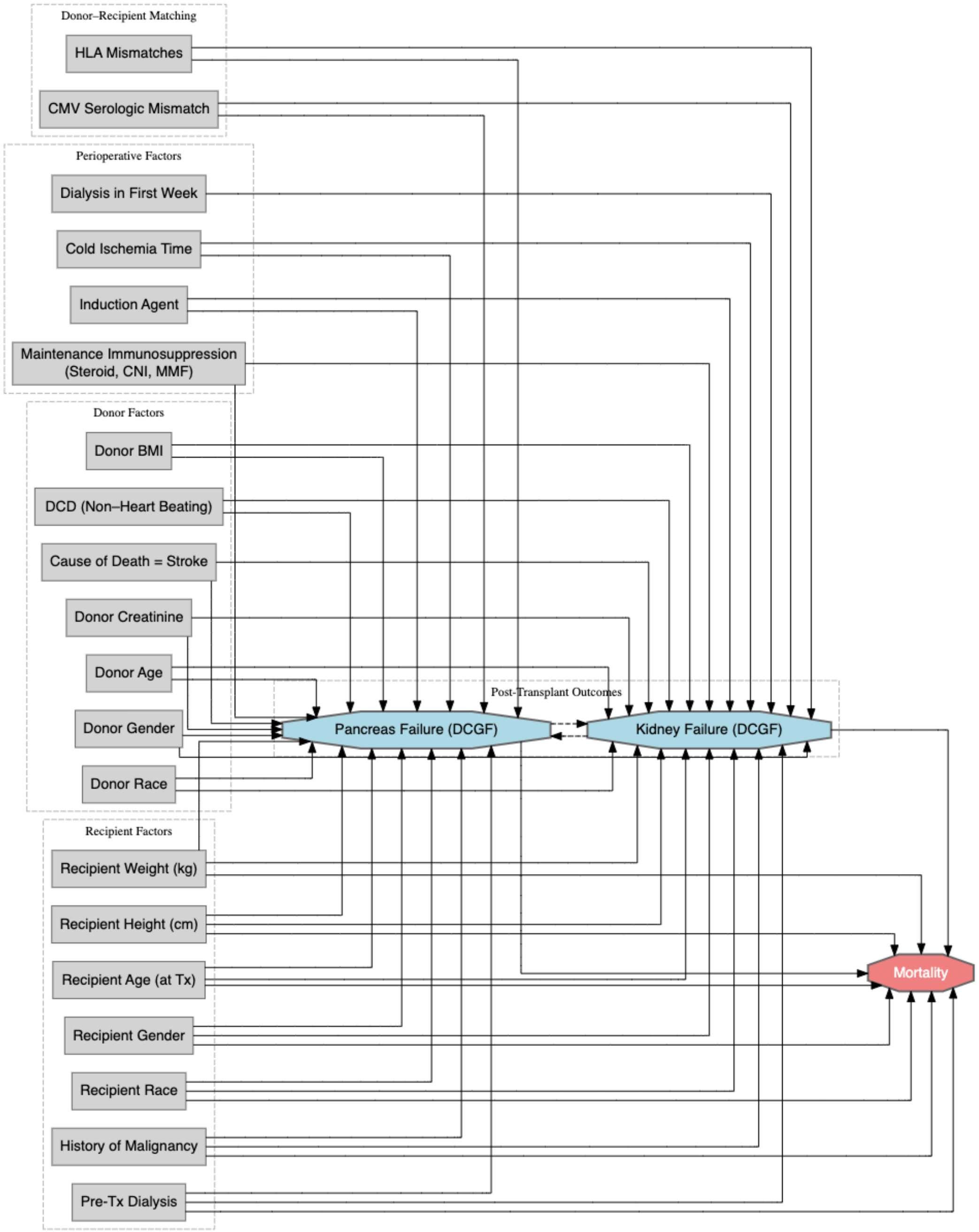

Figure S2. Cumulative incidence of pancreas death-censored graft failure in the overall cohort.

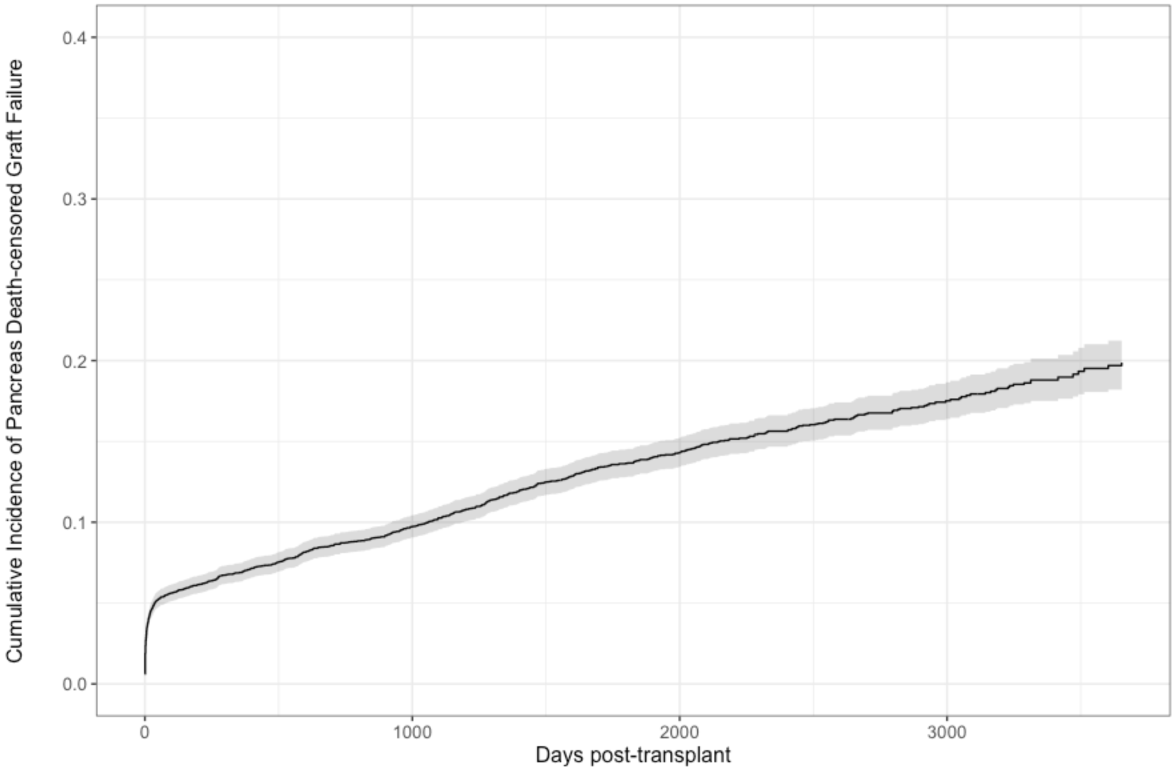

At Risk	7677	5222	2809	977
Events	47	706	916	993

Figure S3. Number of pancreas death-censored graft failure events by (A) post-transplant year; and (B) months in the first-year post-transplant

(A)

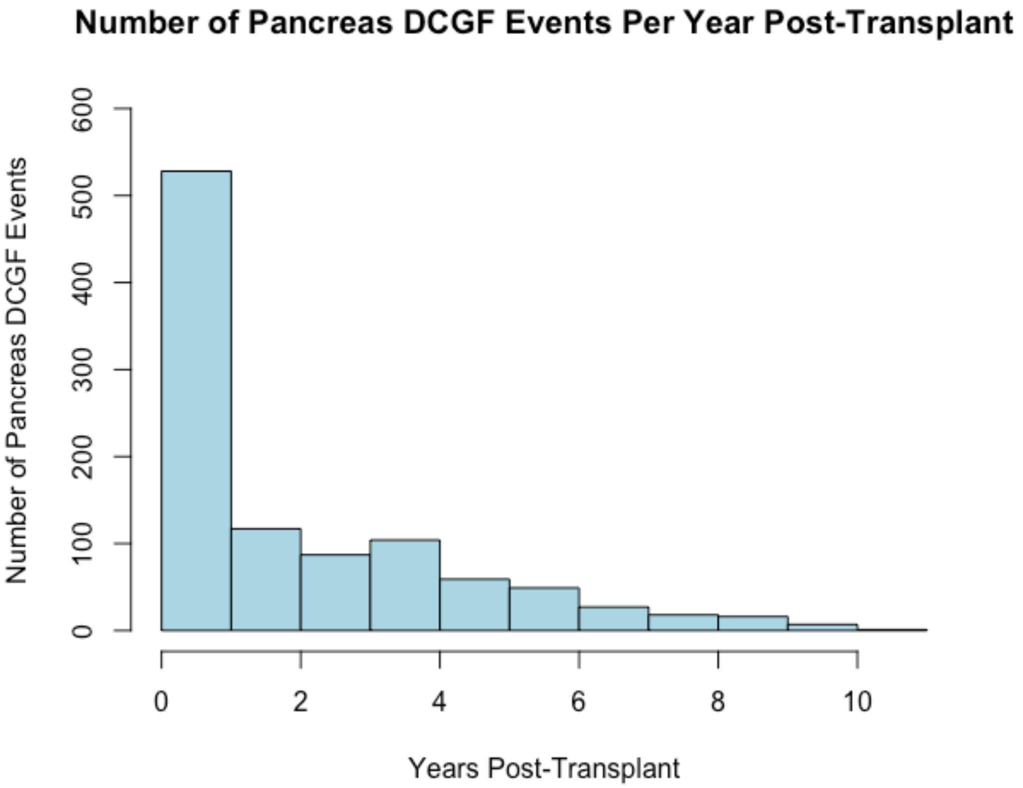

(B)

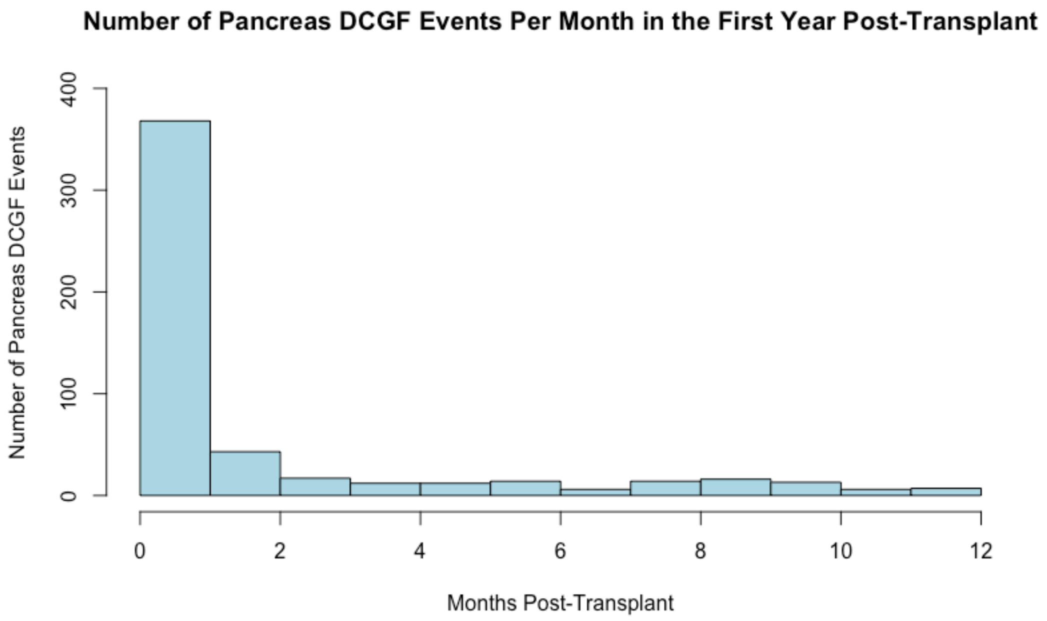

Figure S4. Recipients grouped by graft function for (A) all-comers, and (B) among those with graft failures.

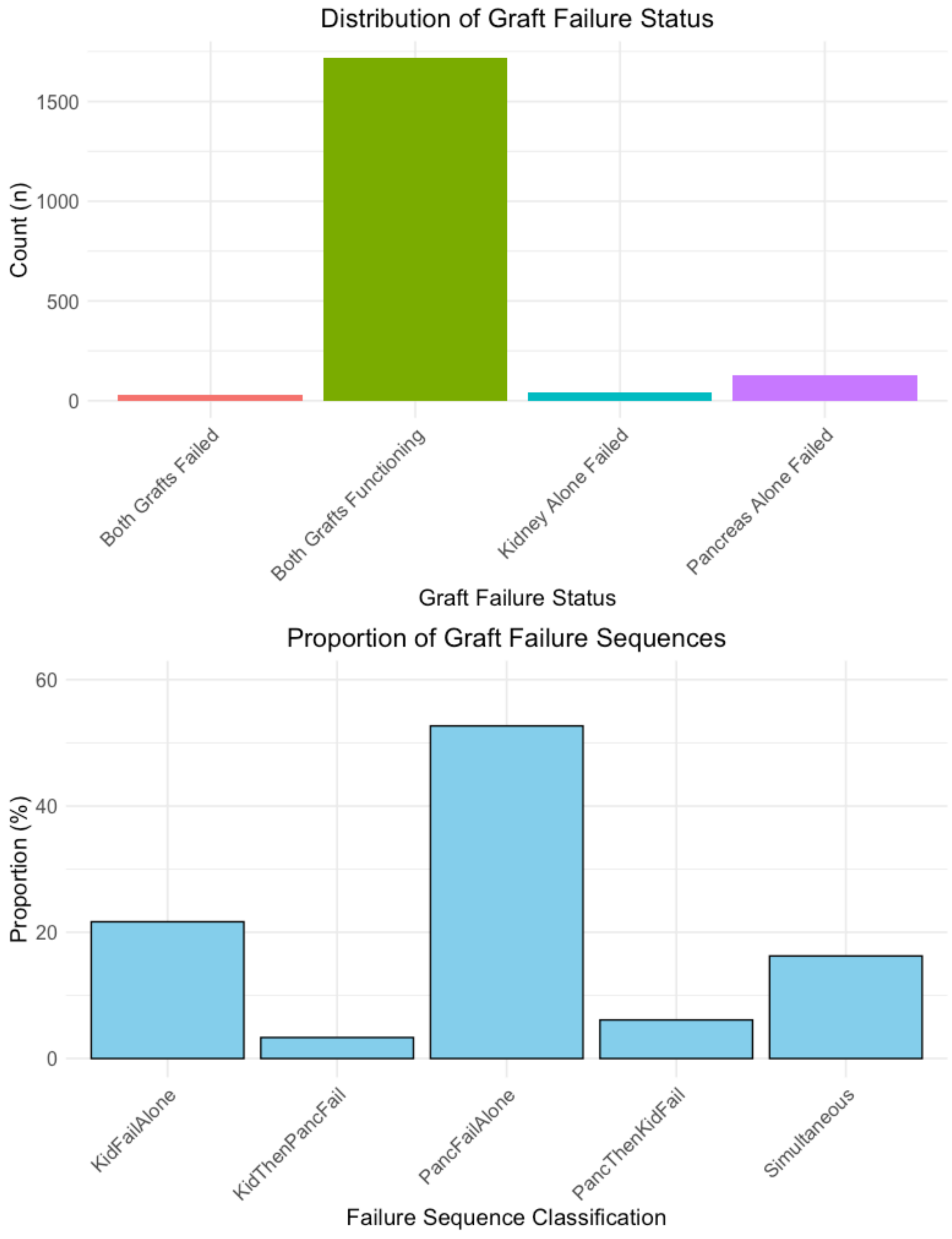

- Both grafts failed: 332
  - Simultaneous failures (within 1 year): 210
  - Kidney failed first: 43
  - Pancreas failed first: 79
- Kidney graft failure alone: 280
- Pancreas graft failure alone: 681
- No graft failure: 6384

Figure S5. Kaplan-Meier Curve Showing All-Cause Mortality from Time of Pancreas Graft Failure (n=1,013 recipients with pancreas death-censored graft failure; 147 deaths)

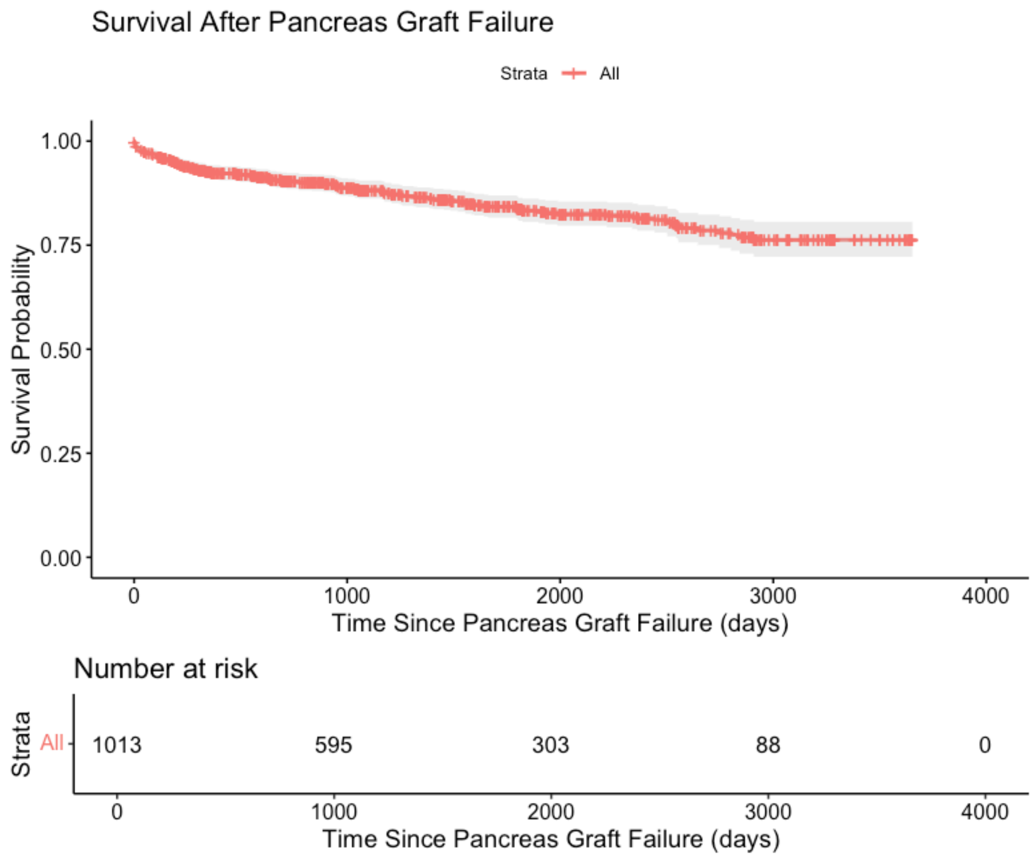

Figure S6. (A) Within-cluster sum of squares across possible number of clusters. (B) Box plot of hazard ratio for pancreas DCGF-related mortality in younger and older age groups, with 95% confidence intervals.

(A)

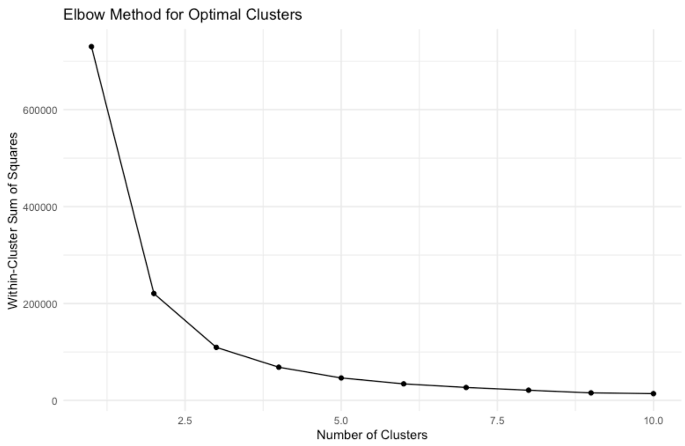

(B)

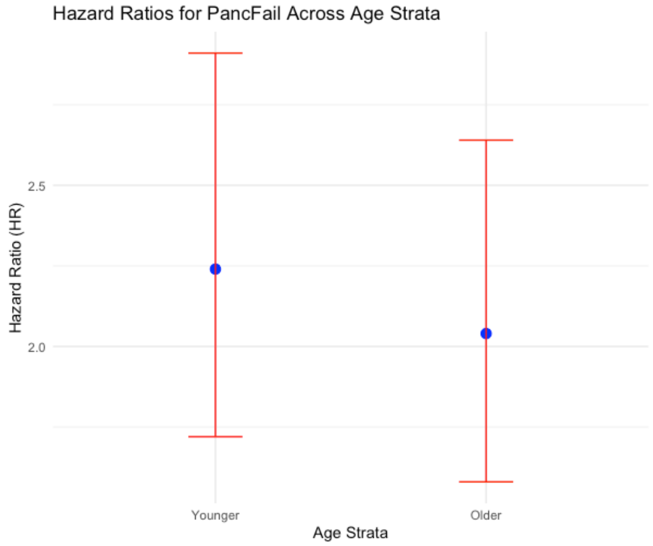

- Significant interaction between pancreas graft failure and recipient age, as predictor of mortality. ANOVA $p<0.001$ comparing models with and without interaction.
- Optimal number of age clusters: 2
  - Younger age: 13-42 yrs. Mean 34.9 yrs. N=4718 pts.
  - Older age: 43-75 yrs. Mean 50.3 yrs. N=3972 pts.
- Older age group:
  - n= 3972, number of events= 443
  - HR 2.04 (95% CI 1.58, 2.64)
- Younger age group:
  - n= 4718, number of events= 326
  - HR 2.24 (95% CI 1.72, 2.91)
- Similar relative hazard ratios for pancreas graft failure-related mortality. Ratio of hazard ratios was 1.10.
