# Supplementary material for: The Time-varying Impact of Pancreas Graft Failure on Mortality and Kidney Graft Outcomes in Simultaneous Pancreas and Kidney Transplantation
Source: Transplant Direct. 2026 Jun 2;12(7):e1942. doi: 10.1097/TXD.0000000000001942 (PMC13232909; doi:10.1097/TXD.0000000000001942)
Supplement: Supplementary file 2 [file txd-12-e1942-s002.pdf]

Table S1. Reasons for pancreas graft failure among those who had death-censored graft failure. (n=1013)

Technical vs non-technical	Subgroup	Number of cases overall	Number of cases within 1 year post-transplant	Number of cases > 1-year post-transplant
Technical (n=334)	Anastomotic leak	29	318	16
	Bleeding	23		
	Graft/vascular thrombosis	282		
Non-technical (n=352)	Hyperacute rejection	1	77	275
	Acute rejection	140		
	Chronic rejection	171		
	Infection	40		
Unknown (n=327)	Other	270	133	194
	Primary non-function	29		
	Pancreatitis	28		

Table S2. Distribution of reported causes of death by pancreas graft failure status

Cause of death type	Pancreas graft failure (%)	No pancreas graft failure (%)
Cardiovascular	24 (16.3%)	114 (18.3%)
Graft failure	5 (3.4%)	9 (1.5%)
Hemorrhage	5 (3.4%)	10 (1.6%)
Infection	16 (10.9%)	53 (8.5%)
Malignancy	7 (4.8%)	32 (5.1%)
External/behavioural	0 (0%)	15 (2.4%)
Other medical	25 (17.0%)	124 (19.9%)
Unknown	65 (44.2%)	265 (42.6%)
• Chi-squared test p = 0.21		
